# Physician workforce in the United States of America: forecasting nationwide shortages

**DOI:** 10.1186/s12960-020-0448-3

**Published:** 2020-02-06

**Authors:** Xiaoming Zhang, Daniel Lin, Hugh Pforsich, Vernon W. Lin

**Affiliations:** 1 Cleveland Clinic Foundation, Neurological Institute, Cleveland, OH United States of America; 2 University of California at Davis, Davis, CA United States of America; 3 California State University, Sacramento, CA United States of America; 4 Physical Medicine and Rehabilitation Service, The Hershel “Woody” Williams VA Medical Center, Huntington, WV United States of America

**Keywords:** Physician workforce, Demand, Supply, Physician shortage, Report card

## Abstract

**Background:**

Physicians play a critical role in healthcare delivery. With an aging US population, population growth, and a greater insured population following the Affordable Care Act (ACA), healthcare demand is growing at an unprecedented pace. This study is to examine current and future physician job surplus/shortage trends across the United States of America from 2017 to 2030.

**Methods:**

Using projected changes in population size and age, the authors developed demand and supply models to forecast the physician shortage (difference between demand and supply) in each of the 50 states. Letter grades were then assigned based on projected physician shortage ratios (physician shortage per 100 000 people) to evaluate physician shortages and describe the changing physician workforce in each state.

**Results:**

On the basis of current trends, the number of states receiving a grade of “D” or “F” for their physician shortage ratio will increase from 4 in 2017 to 23 by 2030, with a total national deficit of 139 160 physician jobs. By 2030, the West is forecasted to have the greatest physician shortage ratio (69 physician jobs per 100 000 people), while the Northeast will have a surplus of 50 jobs per 100 000 people.

**Conclusion:**

There will be physician workforce shortages throughout the country in 2030. Outcomes of this study provide a foundation to discuss effective and efficient ways to curb the worsening shortage over the coming decades and meet current and future population demands. Increased efforts to understand shortage dynamics are warranted.

## Background

Improving quality of care, increasing access to care, and controlling healthcare costs depend on the adequate availability of healthcare providers [1]. Due to aging, population growth, and a greater insured population following the Affordable Care Act (ACA), physician availability to patients has been recognized as one of the top barriers to meet the healthcare needs of patients in the United States of America [2]. For instance, the Bureau of Labor Statistics (BLS) predicts that 91 400 physician jobs will be needed nationally; this is a 13% increase from 2016 to 2026 [3]. Meanwhile, it is predicted that there will be a physician shortage in the next decade because the demand for physicians is growing faster than the supply of physicians [4]. According to the Health Resources and Services Administration’s (HRSA) report in 2011 [5], there was an estimated existing deficiency of 17 722 primary care providers in the United States of America. Furthermore, in 2020, the United States of America may face shortages of 45 400 primary care physicians and 46 100 medical specialists—a total shortage of 91 500 doctors in 2020 [6]. Only in the most optimistic supply and demand scenarios would the nation have an adequate supply to meet demand in the year 2020 [7]. In a recent study, the Association of American Medical Colleges (AAMC) predicted that by 2030, the demand for doctors will outstrip the supply and that the United States of America will experience a shortage of up to 121 300 physicians [8]. The physician shortage is increasing steadily throughout the nation and will influence the delivery of healthcare, thus affecting patient outcomes negatively.

Healthcare First is a health service study group that began its investigation of the distribution of registered nurses (RN) throughout the state of California in 2005 [9, 10]. Since then, the group has expanded its scope to study social workers [11], physical therapists [12], occupational therapists [13], RN for the United States of America [14], and now the physician workforce. This study uses a similar methodology as the previous studies to examine the current surplus/shortage trends in the physician workforce across the United States of America and to make predictions for these trends to the year 2030. It also applies the methodology to each individual state and assigns a workforce grade to each state. This analysis should prove beneficial in the development of policies that address the availability of physicians throughout the United States of America.

## Methods

### Design and sample

This article used the same forecast and grading methodology developed in previous healthcare provider shortage forecast studies [14, 15]. Physician job shortages were projected by investigating the differences between physician demand and physician supply in all 50 states (Table 1, key term definitions). With the use of public databases, a forecasting model was constructed to project the demand and supply of physician jobs in the United States. The combination of these supply and demand models was used to produce physician shortage forecasts for the coming years. A grading methodology was then used to evaluate individual state shortage ratios between 2017 and 2030. In order to analyze the national shortage more specifically, the states were grouped into four regions (West, Midwest, South, and Northeast) as defined by the Bureau of Labor Statistics.

**Table 1:** Explanation of key terms

Key terms	Definition
Bureau of Labor Statistics (BLS)	The official source of labor economic and statistical data for the federal government. Through a semiannual survey, the BLS produces employment and wage estimates for 800 different occupations on the national, state, and sub-regional levels (www.bls.gov).
Centers for Medicare & Medicaid Services (CMS)	Source of age-based personal health care expenditure estimates.
Current Population Survey (CPS)	CPS is a monthly survey of about 50 000 households conducted by USCB and BLS. CPS is the primary source of information on the labor force characteristics of the US population.
Report card	A collection of grades assigned to each state based on a grading rubric used for determining stated (20011) or projected (2030) physician shortage ratios.
National mean	195 Physician jobs per 100 000 people. This value was based on the number of physicians in the United States of America per 100 000 people for 2011.
Personal health care expenditure (PHE)	An estimate that takes into account “spending for hospital care, physician and clinical services, dental care, other professional services, home healthcare, nursing home care, and healthcare products purchased in retail outlets.” This estimate does not include spending on public health programs, health facility administration, healthcare research, and the construction of healthcare facilities (Centers for Medicare and Medicaid Services, 2018).
Physicians (including surgeons)	Physicians diagnose and treat injuries and illnesses in patients. Physicians examine patients, take medical histories, prescribe medications, and order, perform, and interpret diagnostic tests. Surgeons operate on patients to treat injuries, such as broken bones; diseases, such as cancerous tumors; and deformities, such as cleft palates.
Physician jobs	A worker who can be classified as a full-time or part-time physician. This is the fundamental unit of measure used to estimate physician populations and is counted through a survey conducted by the BLS every 3 years.
Physician demand	The estimated number of physician jobs needed to meet population needs.
Physician demand ratio	The number of physician jobs needed per 100 000 people.
Physician shortage	The difference between a region’s demand for physician jobs and that region’s supply of physician jobs.
Physician shortage ratio	Physician shortage per 100 000 people.
Physician supply	The estimated number of physician jobs.
Physician supply ratio	The number of physician jobs per 100 000 people.
US Census Bureau (USCB)	USCB is a government agency that is responsible for the US Census. USCB is responsible for collecting and providing relevant data about the people and economy of the United States of America.

### Demand model

The demand model was based on the previous model with updated values. In order to find the demand, the study team utilized numbers published by the Centers for Medicare & Medicaid Services (CMS) about age-based personal health care expenditure (PHE) estimates for 2010 [16]. Age-population projections from the United States Census Bureau (USCB) [17] were used with the age-based PHE estimates to forecast future demand for health services until 2030 as a single dollar amount. Using linear regression analysis, the nation’s healthcare expenditure was plotted against the BLS-reported number of physician jobs nationally from 2004 to 2017. This resulted in a slope of 4.14 × 10^−7^(*R*^2^ = 0.963). This slope was used to convert change in PHE to change in physician jobs for the nation and each state. The equation for the demand model is as follows:

$$ {D}_{R,N}=203\times \left[2017\ \mathrm{Projected}\ \mathrm{State}\ \mathrm{Population}\right]/1{0}^5+4.14\times 1{0}^{-7}\times \left(\varDelta {\mathrm{PHE}}_{R,2017,2018}+\varDelta {\mathrm{PHE}}_{R,2018,2019}+\dots +\varDelta {\mathrm{PHE}}_{R,N-1,N}\right) $$

where *D* is the demand, *R* is the region or state, and *N* is the year and ΔPHE_*R*,*N*−1,*N*_ = PHE_*N*_ − PHE_*N*−1_. The number 203 is the national mean of physician jobs (physician jobs per 100 000 people); 4.14 × 10^−7^is the linear slope of change in PHE to the number of physician jobs.

### Supply model

The propensity or probability of a US citizen to work as a physician was calculated using estimates provided by the Current Population Service about the physician age-population [18]. Physician population estimates were collected over the course of 14 years from 2004 to 2017 in the following seven age groups: 16 to 19, 20 to 24, 25 to 34, 35 to 44, 45 to 54, 55 to 64, and 65 and older. These numbers were then divided by the population in the same age groups which yielded the physician propensity. The following formula contains details of the supply model:

$$ {S}_{R,N}={\mathrm{BLS}}_{2017}+{\sum}_R\left({L}_A\times \left({\Delta \mathrm{POP}}_{A,2017,2018}\right)+{\sum}_R\right({L}_A\times \left({\Delta \mathrm{POP}}_{A,2017,2018}\right)+\dots +{\sum}_R\Big({L}_A\times \left({\Delta \mathrm{POP}}_{A,N-1,N}\right) $$

where *S* is the supply, *R* is the region or state, *N* is the year, *L* is the likelihood averaged over 14 years, and *A* is the age group; ΔPOP_*A*,*N*−1,*N*_ = age group-specific Population_*N*_ − Population_*N*−1_; and BLS_2017_ is the number of physician jobs reported by the BLS in 2017.

### Report card

The method used to determine grading in this article is the physician shortage, which is the difference between the physician demand and the physician supply per 100 000 people, as shown in the following equation.

$$ \frac{\left[\mathrm{State}\right]\ \mathrm{Physician}\kern0.17em \mathrm{Demand}-\left[\mathrm{State}\right]\;\mathrm{Physician}\kern0.17em \mathrm{Supply}}{\left[\mathrm{State}\right]\;\mathrm{Total}\kern0.17em \mathrm{Population}}\times {10}^5=\left[\mathrm{State}\right]\;\mathrm{Physician}\kern0.17em \mathrm{Shortage}\kern0.17em \mathrm{Ratio} $$

The national and state numbers of physician jobs in 2017 were retrieved from the BLS [19]. Population projections were obtained from the USCB [17]. The report card was based on the national physician supply ratio, or national mean, of 203 physician jobs per 100 000. This value served as the standard value for comparison for state performances in the years to come. The standard deviation (SD) of the physician supply ratios across the 50 states formed the framework of the grading rubric (SD of 50 states is 57 physicians per 100 000 people in this study). Letter grades were given based on the difference between the national mean and each state’s shortage ratio with the national mean serving as the “C” grade. A and F grades were given for physician job shortage ratios ± 2 SD from the mean, B and D were ± 1 SD from the mean, and C+ and C− were ± 0.5 SD from the mean.

## Results

**Fig. 1:**
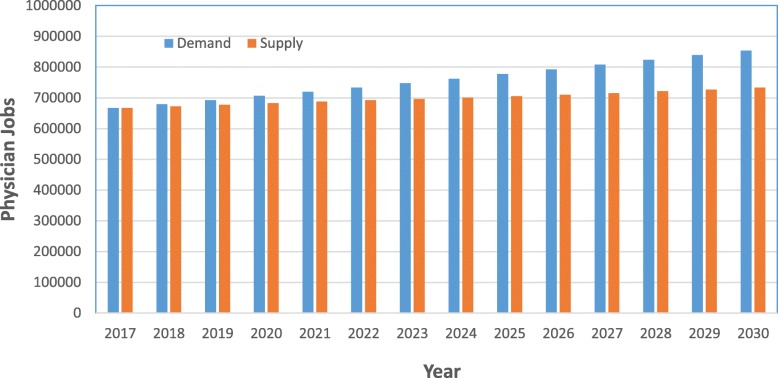
Projected physician demand and physician supply for the United States of America from 2017 to 2030

This study breaks down physician shortages into three different levels: national, regional, and state. Nationally, physician shortages will continue to grow across the country through 2030 (Fig. 1). The United States of America will face an estimated shortage of 139 160 physicians by 2030, and this significant shortage will have varying impacts on each region. Among the four regions, those with the largest estimated shortage in 2030 will be the South (92 172 jobs) and the West (63 589 jobs); the Midwest will have a lower shortage of 16 291 jobs. The Northeast is the only region predicted to have a surplus of physician jobs with an excess of 28 627 jobs. In terms of physician shortage ratios in 2030, the West is forecasted to have the greatest shortage (69 physician jobs per 100 000 people) followed by the South with 62 physician per 100 000 people. The Midwest will have a shortage ratio of 41 jobs per 100 000, and the Northeast will have a surplus of 50 jobs per 100 000.

On the state level (Table 2), the states with the greatest estimated physician shortage will be California (32 669 jobs), Florida (21 978 jobs), and Texas (20 420 jobs). In terms of shortage ratio (physician shortage per 100 000 people), the states with the largest shortage ratio will be Mississippi (120), New Mexico (101), and Louisiana (100). The states with the least shortage in terms of shortage ratio are Massachusetts (− 145), Vermont (− 95), and New York (− 76). Each state shows an increase in shortage ratio ranging from 16 to 57 people per 100 000 when comparing the data between 2017 and 2030 (Table 2). States with the largest increase in shortage ratio will be New Mexico (57), Wyoming (57), and Delaware (54). With regard to grades, in 2017, there were two As, five Bs, seven C+s, 19 Cs, 13 C−s, four Ds, and 0 F. Only Massachusetts and Vermont had an A grade. In 2030, there will be one A, three Bs, two C+s, 10 Cs, 11 C−s, 22 Ds, and 1 F with Massachusetts being the only one having a grade of A. Using a numeric grading scale in which A = 4, B = 3, C+ = 2.33, C = 2, C− = 1.67, D = 1, and F = 0, the national grade point average was 2.06 in 2017, a C grade average. By the year 2030, this national grade point average is expected to decrease to 1.56, a C− grade average.

**Table 2:**
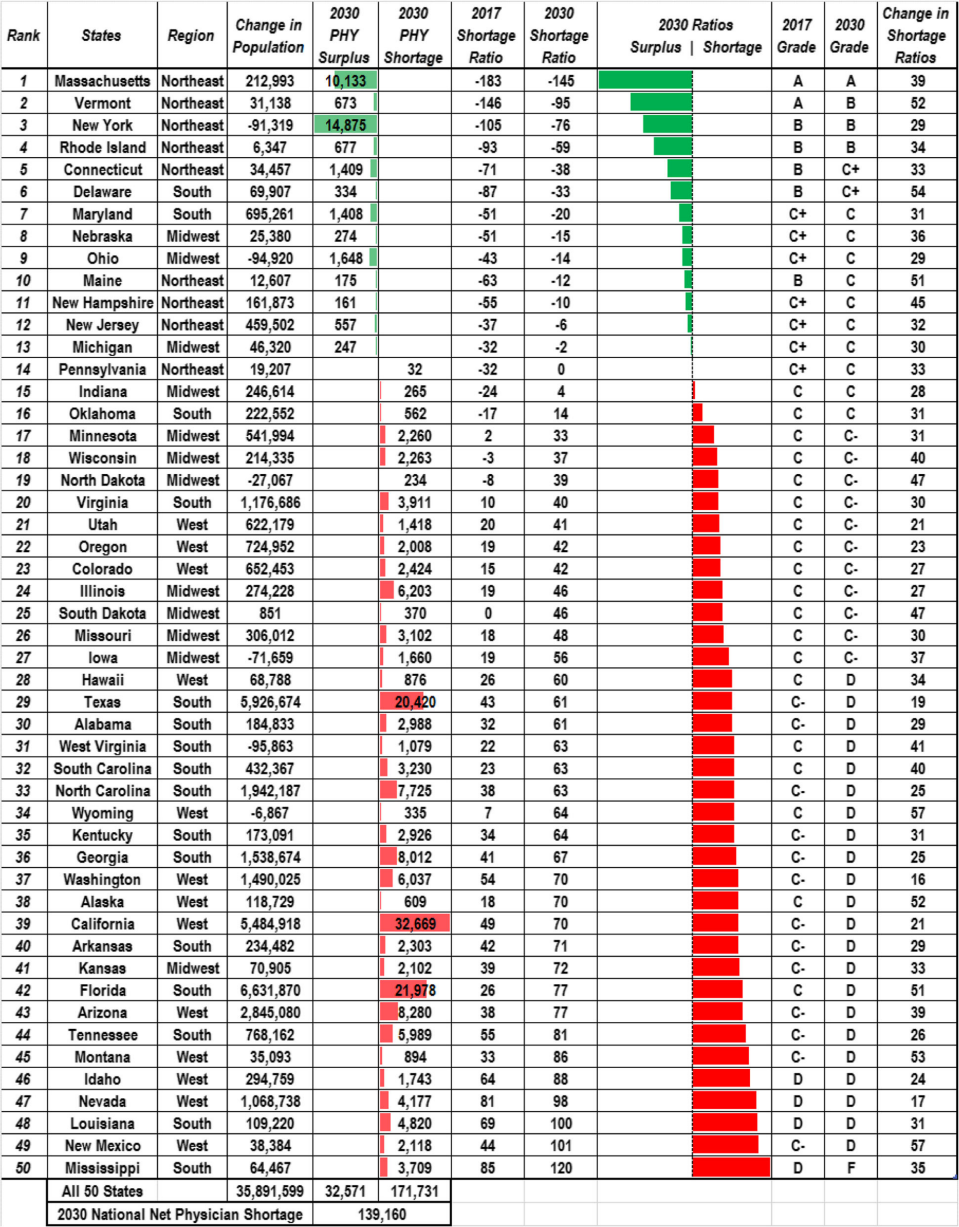
States organized by BLS-defined region and the change in physician-related factors for 2017–2030

## Discussion

**Fig. 2:**
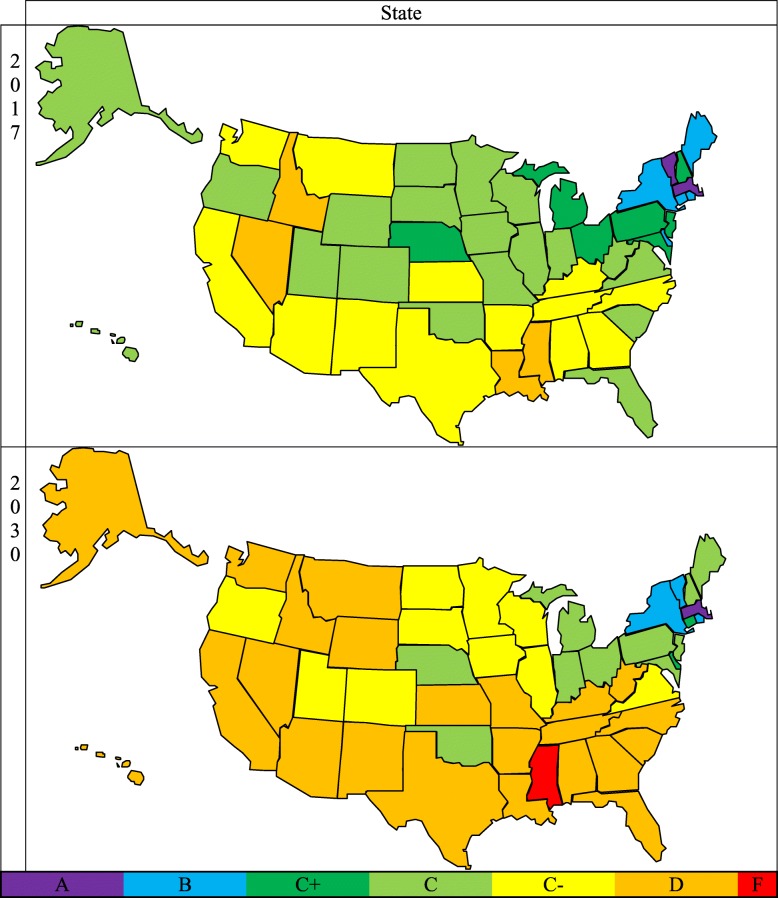
National grade distribution in 2017 (top) and 2030 (bottom). The results show that there are currently physician shortages in some states, and these shortages are forecasted to increase through the year 2030

This study predicts that the demand for physician services will significantly outpace the supply of physicians within the United States of America from 2017 to 2030, causing many states to face severe physician workforce shortages (Fig. 2). By 2030, 34 out of 50 states will have physician shortages with a grade of C− and below. Most of these states are located in the South and West regions, which is consistent with our prior publications regarding the shortage of nurses [14, 15].

Examination of the healthcare workforce is difficult due to the complexity of factors (e.g., population growth, age, economics, healthcare policy, healthcare practice, geography, models of care, new technologies, and innovations) that affect supply, demand, and balance between supply and demand in each state. By 2030, the following states will have the largest physician shortages (the number of physician jobs): California (32 669 jobs), Florida (21 978 jobs), and Texas (20 420 jobs). The key reasons for high shortages in these three states are attributed to high physician demand from the growth of the total population, aging state population, and aging physicians without an adequate commensurate increase in physician supplies. Taking California as an example, the state population and elderly population will grow by 112% and 148% between 2017 and 2030, respectively. Meanwhile, 33.4% of all active physicians in California are over 60 years old and within 5 years of retirement, although at the national level, only 30.3% of all active physicians are 60 years old and above [20]. From 2006 to 2016, the number of medical students at Doctor of Medicine (MD) and Doctor of Osteopathic Medicine (DO) schools increased by 20.7% (7387 students), and the number of residents and fellows increased by 12.8% (10 429 students) [20]. On the basis of current trends, the number of new licensees per year is not large enough to replace these physicians as they retire, and physician demand will outpace the physician supply within the state of California [21].

Florida and Texas, the two other states with the largest physician shortage, are experiencing similar situations as California. These two states are on the top of the list of the fastest-growing states in the United States of America. In the next 10 years, Florida’s population will increase by 30% and Texas will increase by 22%. Furthermore, by 2030, the aging population will increase 73% and 55% in Florida and Texas, respectively. Physicians are aging along with the general population in these two states, which will have a profound impact on the physician workforce. Approximately, 34.3% of all active physicians in Florida [22] and 27.2% of all active physicians in Texas [23] are over 60 years old. It should be noted that Florida has the seventh oldest physician population in the United States of America [22] with a total of 16.6% of its physicians planning to retire in the next 5 years [24]. In the past 10 years, these two states rapidly expanded their number of medical students, residents, and fellows. From 2006 to 2016 in Florida, there was a 70.9% increase in the number of medical students and a 50.5% increase in the number of residents and fellows. The percentage changes in Texas are 31.6% and 18.6%, respectively [20]. It must be taken into account that the growth of medical students outpaces the growth of residents and fellows. Thus, continual efforts to increase the pipeline of physicians in these states are paramount.

As for physician shortage ratios (physician jobs per 100 000 people), by 2030, the three states with the most severe physician shortage ratios will be Mississippi (120), New Mexico (101), and Louisiana (100). The only state expected to have an F grade is Mississippi. Mississippi has a low physician availability of 118 active physicians per 100 000 people, 42% below the national mean of 203. Consequently, Mississippi will require an additional 3709 physicians by 2030, a 51% increase of the state’s current 3528 practicing physicians (as of 2017) to meet the national benchmark. Since 2006, the MD and DO schools in Mississippi have increased their enrollment by more than 130%, which ranked first in percentage change nationally. By 2022, Mississippi medical school graduates will likely increase to more than 265 students annually, which is more than 2.5 times the Mississippi medical student graduation rate prior to 2005 [25, 26]. In addition, Mississippi increased its graduate medical education (GME) first-year training positions at community-based GME sites from 14 to 56 and its residency slots from 42 to 108 [25]. These actions can help Mississippi to train and retain more medical professionals within the state to mitigate its physician shortages.

New Mexico has the second-worst projected shortage ratio and is also the state with the largest change in shortage ratio, rising 57.1 physician jobs per 100 000 people by 2030 (Table 2). Among most states, aging of the physician workforce is a significant factor contributing to future state shortages. New Mexico has the oldest physician workforce in the nation, with 37% of physicians over 60 years old and facing retirement in the next 10 years [27]. To maintain the status quo, New Mexico will require an additional 2118 physicians by 2030, a 40.4% increase of the state’s current 3128 physicians (as of 2017). The aging physician population reflects the difficulty New Mexico has in attracting and retaining young physicians.

Massachusetts and Vermont are the two states with the highest physician surplus. Both of these two states are located in the Northeast region, where GME training programs have historically been located. The distribution of residents is particularly important, given evidence that physicians tend to practice in geographic areas similar to those where they complete their GME training. Every year, federal GME spending of over $15 billion trains residents across the country [28]. The Northeast region received $5.47 billion (38%) of total federal spending, which is almost three times what the West received ($1.83 billion, 13%). Consequently, 31% of GME residents were located in the Northeast [28]. It should be noted that the states with a physician surplus (graded A or B) will face increasing pressures from states with physician shortages (graded C− and below) to attract and retain physicians from their state [29].

There are a few related studies in the literature which examine the status of the physician workforce. HRSA produced a model of patient demand for primary care services that also incorporated the sizable challenges of an aging and growing population [30]. This study projected a 23 640 primary care physician shortage in 2025. With delivery system changes and full utilization of mid-level healthcare providers, including nursing practitioner (NP) and physician assistant (PA) services, the projected shortage of 23 640 primary care physicians can be effectively mitigated. More recently, AAMC published a report on the supply and demand of physicians projected through 2030 [8]. This study supports the notion that an increasingly older population will result in an increase in the demand of physicians and cause a greater shortage of physicians. Using multiple different scenarios to reflect different assumptions, such as the use of NPs and PAs to assist staffing problems, AAMC predicted that the physician shortage will be between 42 600 and 121 300 in 2030. Based on our models, we predict that there will be a shortage of 72 472 physicians in 2025 and 139 160 physicians by 2030. These findings are consistent with the aforementioned reports under the scenario without mid-level healthcare services. In many states, mid-level healthcare providers are present to mitigate the shortage of physicians. With the assistance of these services, the healthcare industry is able to utilize a new patient care model that is more reliant on a team-based care delivery in order to care for an increasing number of insured and elderly patients. This team-based care model helps healthcare organizations work together more effectively and efficiently [31]. The utilization of mid-level healthcare providers may help to mitigate the physician shortage, it cannot completely replace physicians [32].

Recently, the New York University School of Medicine announced that it would eliminate tuition to encourage people to pursue medical careers [33]. Without the prospect of overwhelming financial debt, more people will pursue medical careers. People view this decision as a positive step forward, but they also caution that it might not be a silver bullet for America’s worsening physician shortage. Presently, the United States of America is not facing a medical student shortage, but rather, a residency shortage. Because of the 1997 cap on Medicare to support GME, the necessary commensurate increases in residency training have been stymied [34], creating a bottleneck for the physician supply. In 2018, there was a record-breaking 37 103 US and international medical school students and graduates competing for only 33 167 positions, a shortfall of about 4000 residents-to-be [35]. To address this issue, the Resident Physician Shortage Reduction Act of 2017 (H. R. 2267) was introduced in Congress to increase by 3000 the annual number of residency slots from 2019 to 2023 [36]. States such as Arkansas, Kansas, Missouri, and Utah have also passed legislation to provide provisional licenses to some medical school graduates who have not been able to find residency spots [37]. With these provisional licenses, they can practice primary care under the medical license of another physician, but only in medically underserved areas. Some institutions have also created their own medical-school-to-residency pathways. For example, Kaiser Permanente currently trains 600 residents annually and provides continuing medical education (CME) to another 22 000 medical professionals [38].

With the rapidly increasing demand for physicians, many US healthcare institutions turn to foreign-trained doctors to supplement their physician workforce [39]. Foreign-trained doctors have long been an integral part of the US healthcare system, contributing substantially to primary care disciplines and providing care in underserved populations. According to the Organization for Economic Cooperation and Development [40], in 2016, there were more than 215 630 foreign-trained doctors practicing in the United States of America, a number far surpassing any other country. It should be noted that the global physician supply is finite and the competition for these providers exists across national boundaries [41].

As technology evolves, its impact on the medical field increases. For example, artificial intelligence (AI) algorithms will shift the roles of physicians from a knowledge-based role into more of a skills-based role [42]. All of that information will be available in an AI-driven database that can not only bring up the information at a moment’s notice, but also help to diagnoses. Technology enables physicians to spend less time testing samples and recording data and spend more time providing quality care to their patients. Technology also helps medical institutions operate more effectively and efficiently, which may alleviate some of the burdens of the physician shortage.

There were several limitations to our study involving the construction of demand and supply models. We made a crucial assumption in the demand model by using the current (2017) national ratio of physicians to the overall population (203 physicians per 100 000 people) as a baseline. We assumed that no shortage exists in this ratio, since a relative zero value was needed to calculate future shortages. This study does not comment on whether the country currently lacks physicians, but asserts that shortages are imminent, based on current trends in supply and demand. Therefore, if our assumption that the 2017 baseline without shortage is incorrect, our projections may underestimate the true nature of the shortage. In addition, the national slope was used in converting change in PHE to physician jobs to avoid state variations in physician workforce responsiveness to health expenditures. Further analysis indicated that change in PHE translates to a larger change in physician demand in some states than represented by the national slope. The responsiveness of physician jobs to PHE also may vary depending on the work setting for physicians.

The primary assumption in the supply model is that the average likelihood or propensity of an individual to choose to be a physician at a certain age is the same across every state and will be the same in the coming years. This does not address individual states’ differences in their ability to recruit young people into the medical profession and the capacity of their medical schools, including the number of faculty present. Moreover, we used an average propensity value over the past 10 years, which does not account for the increasing enrollment rates in medical schools. If enrollment continues to increase as it has for the past decade, the physician propensity value for those between ages 21 and 34 will be an underestimation. Another limitation that may underestimate the physician supply is the exclusion of future arrivals of foreign-born physicians. According to a study in 2015, almost a quarter of residents across all fields, and more than a third of residents in sub-specialist programs were foreign medical graduates [39]. This means that we are reliant on physicians trained outside the country to fill the gap.

## Conclusion

The results in this study suggest that physician shortages currently exist in many states across the nation and will likely increase over the next 10 years and may influence the delivery of healthcare, negatively affecting patient outcomes. Steps have been taken to prepare the physician workforce to meet the growing demand for health services, which include rising numbers of medical school graduates, attracting foreign-trained doctors, utilization of mid-level providers, and application of emerging technology. We hope that the information derived from this study can guide future workforce research and inform health workforce planners, employers, educators, and policy-makers regarding the development of concrete national, regional, and/or state strategies to reduce physician shortages.

## Data Availability

The datasets used and/or analyzed during the current study are available from the corresponding author on reasonable request.

## Abbreviations

AAMC: Association of American Medical Colleges
ACA: Affordable Care Act
AI: Artificial intelligence
BLS: Bureau of Labor Statistics
CME: Continuing medical education
CMS: Centers for Medicare & Medicaid Services
DO: Doctor of Osteopathic Medicine
GME: Graduate medical education
HRSA: Health Resources and Services Administration
MD: Doctor of Medicine
NP: Nursing practitioner
PA: Physician assistant
PHE: Personal health care expenditure
RN: Registered nurses
SD: Standard deviation
US: United States
USCB: United States Census Bureau (USCB)
